# Prevalence of Non-Nutritive Sucking Habits and Potential Influencing Factors among Children in Urban Communities in Nigeria

**DOI:** 10.3389/fped.2015.00030

**Published:** 2015-04-20

**Authors:** Adebola Emmanuel Orimadegun, Gabriel Omen Obokon

**Affiliations:** ^1^Institute of Child Health, College of Medicine, University of Ibadan, Ibadan, Nigeria

**Keywords:** nipple dummy, finger sucking, acute diarrhea, pacifiers

## Abstract

**Background:**

The use of non-nutritive sucking materials like pacifiers and fingers poses health challenges to children in resource-limited settings, where hygiene practices and provision of clean water are poor. This study was designed to determine the prevalence of non-nutritive sucking habits and its association with acute diarrhea in children aged 6–23 months in urban communities of Nigeria.

**Methods:**

In this cross-sectional study, 12 communities from 4 out of 12 geopolitical wards in Ibadan North Local Government Area and 427 mothers of children aged 6–24 months were randomly selected. A pre-tested, interviewer-administered questionnaire was used to obtain information on socio-demographic characteristics, recent history of diarrhea (3 months prior to visit) and use of non-nutritive sucking materials. Descriptive statistics, Chi-square, and logistic regression were used for data analysis at *p* = 0.05.

**Results:**

Mean age of the children was 13.9 ± 5.3 months and 50.6% were males. Prevalence of non-nutritive sucking was 45.2%. Prevalence of non-nutritive sucking was not significantly different between males (45.8%) and females (44.5%). The odds ratio of engaging in non-nutritive sucking increases by 6.0% with increasing age (OR = 1.06; 1.02; 1.10). More children who were not exclusively breastfed (53.5%) than exclusively breastfed (26.2%) were likely to engage in non-nutritive sucking (OR = 3.25; 95% CI = 2.07, 5.12). Acute diarrhea was more frequently reported in non-nutritive sucking group than the other (OR = 1.51; 95% CI = 1.03, 2.22).

**Conclusion:**

Non-nutritive sucking was linked with failure to practice exclusive breastfeeding, worse with increasing age, and predisposes to acute diarrhea. Further studies are necessary to verify the nature of these associations.

## Introduction

Non-nutritive sucking habit is the use of natural or artificial nipple-like (pacifier) objects (on which the children suck), for the purpose of “comforting, soothing, or calming the infants and even alleviating the burning and itching of the gums” (1). Often, a pacifier, dummy, or soother is a rubber, plastic, or silicone nipple-like object given to an infant or other young child to suck upon. Some mothers encourage non-nutritive sucking habit as a means of satisfying their children’s unending desire to suck the breast. Children also devise other ways of soothing or comforting themselves via non-nutritive sucking habits such as thumb and digits sucking (physiological pacifiers). Non-nutritive sucking habit is a societal norm in many countries (1) and may be related to a variety of inter-related factors including mother level of education, cultural, and socio-economic factors. These factors play major roles in influencing mothers and caregivers in the decision to introduce pacifier to children (2–4).

Many of the pathogens implicated in the etiology of prevailing illnesses, such as acute diarrhea, enter the body via oral route and children living in unhygienic environments are potentially vulnerable. This vulnerability has been attributed to habitual non-nutritive sucking habits (5, 6). For instance, acute diarrhea is one of the leading causes of child mortality in developing countries including Nigeria and it has been linked with infectious agents acquired via oral route (5, 7, 8). The most recent data from the Nigerian Demographic and Health Survey (NDHS) showed that 10% of children <5 years had diarrhea episode in the 2 weeks before the survey and about 2% passed blood in their stool (9). Thus, the need to continue to search for factors that may be targeted for reducing the prevalence of diseases transmitted via oral route among children was emphasized.

Though non-nutritive sucking habits may be beneficial, the use of pacifiers and nursing bottles in communities where exclusive breastfeeding policy has been implemented are discouraged (10). Some studies have demonstrated reduction in the risk for sudden infant death syndrome (SIDS) associated with non-nutritive sucking (11) but such benefits have been scarcely reported from urban communities in Africa. Also, pacifiers and finger-sucking habits were associated with decreased sucking on the mothers’ breast (12–14) and acute otitis media (15–17). Though anecdotal observations showed that some mothers use pacifiers as replacement for breast nipple sucking during weaning while others do it for social reasons in Nigeria, reports on its prevalence and potential influencing factors are scarce.

Whatever are the reasons for non-nutritive sucking habits, it is clear from literature that there are more adverse health effects than gains. It has been shown that colonization of pacifier surface by infectious microbes does occur and this portends a high risk for gastrointestinal infections including those responsible for acute diarrhea diseases in children (18–20). In situations where good sanitation and hygiene practices cannot be guaranteed, the risk of contamination of non-nutritive sucking materials by infectious microbes potentially put children at risk of infections. However, contrary to the theory of microbial contamination, community-based cross-sectional (6) and hospital-based cohort (21) studies in Brazil, reported no association between pacifier use and community-acquired diarrhea. These authors suggested that in contaminated environments the added risk of using pacifiers would not significantly change the incidence of diarrhea. The fact that the supply of clean water for the purpose of cleansing is better in such community than similar ones in Nigeria limits the extrapolation of such findings. Nigeria still has a high rate of infant and child mortality due to diarrhea and it is believed that multiple risk factors contribute synergistically to the spread of diseases (9). A better understanding of factors influencing the use of pacifiers and the habit of thumb/finger sucking in the Nigerian communities may enhance the design of interventions to reduce the risk of infections transmissible via oral route in children. Therefore, this study was carried out to determine the prevalence of non-nutritive sucking habits and its association with acute diarrhea in children aged 6–23 months in urban communities of Nigeria.

## Materials and Methods

### Study design, area, and population

In this community-based cross-sectional study, we interviewed 427 mothers of children aged 6–23 months. They were recruited from the 12 geopolitical wards in Ibadan North Local Government Area (IBNLGA), Nigeria. The IBNLGA covers an area of 27 kg^2^ and it has a population of 306,795 (census 2006). The IBNLGA was purposefully selected because it has relatively more urban communities than other local government areas in Ibadan. The study was focused on mothers of children 6–23 months who are mainly the Yoruba-speaking people of south west of Nigeria.

### Sample size determination and participants recruitment

The investigators estimated that children aged 6–23 months could be as many as 2,000. It was also assumed that 50% of them would engage in non-nutritive sucking habits, thus the minimum sample size required to achieve the main objective of the study at 95% level confidence with an allowable error rate of 5% was 384. Adjusting for a non-response rate of 10%, this number was increased to 422. However, a two-stage sampling technique was used to randomly select 25 communities and 427 eligible mothers. Eligibility was defined as having a child aged 6–23 months and residence in the urban area of IBNLGA.

### Questionnaire and data collection

A pre-tested semi-structured questionnaire with some items adapted from a previous study (3) was used for data collection. The questionnaire items included age, religion, occupation, educational level, and socio-economic status, conventional pacifier, thumb sucking, digital sucking, sanitation, hygiene practices, breast feeding duration, and complementary feeding practices and acute diarrhea within 3 months prior to interview. The questionnaire was translated into the local language (Yoruba). Diarrhea was defined as the passage of three or more abnormally loose stools per day by a child (22). The contents of the questionnaire were discussed with child health experts in the University of Ibadan and pre-tested among 60 mothers in another local government area (Cronbach’s α = 0.87).

### Data analysis

Data were entered and analyzed using Stata SE 12.1 (StataCorp, TX, USA) statistical software. The main outcome variable was acute diarrhea within 3 months prior to the interview. Independent variables included demographics and non-nutritive sucking habit. Chi-square test was used for test of associations and logistic regression was done to identify predictors of acute diarrhea at *p* = 0.05.

### Ethical consideration

The study protocol was reviewed and approved by the University of Ibadan/University College Ethical Review Committee (Approval number: UI/EC/14/0211). Participation in the study was completely voluntary and written informed consent was obtained from each respondent.

## Results

### Socio-demographic characteristics

The mean age of mothers and their children was 29.7 ± 5.6 years and 13.9 ± 5.3 months, respectively. The sex distribution of the children showed that 216 were males while 211 were females. Other demographic characteristics of the study participants and their associations with non-nutritive sucking habits were as shown in Table 1. The majority (97.4%) of the children were from homes where parents were “married and living together” while few (1.7%) had single parents. Those from families with ≤4 children were 95.8. Almost two-thirds (64.2%) of the children were in the range of second to fourth born of the mothers, 31.6% were first born and others were beyond the fourth position (4.2%) in birth order. About 60.0% of mothers had secondary education, while 22.0% had below secondary education and 18.0% had higher education. The distribution of respondents by the types of toilet facilities, source of drinking water, and household source of water revealed for domestic uses showed that 96.1% had toilet facilities, which included open pit latrine (42.6%), flush toilet (32.3%), and pit latrine with slabs (21.2%). Others reported that they defecate in the near-by bush/field (4.9%). Concerning sources of drinking water, families were using sachet water (45.0%) and borehole water (35.8%). A small percentage used bottled water (14.8%), pipe-borne water (2.3%), and well water (2.1%). Only a few mothers reported that “they treat the water before giving it to their children”; water treatments mentioned included boiling (17.8%), addition of bleaching agent or chlorine (1.2%), and filtering (0.5%).

**Table 1:** **Socio-demographic characteristics and non-nutritive sucking habit**.

	Non-nutritive sucking				OR (95% CI)	*p*
	Users		Non-users	
	*n*	%	*n*	%	
**Parents marital status**
Married living with spouse	189	45.4	227	54.6	1.20 (0.17, 8.61)	0.855
Single parents	2	28.6	5	71.4	2.50 (0.19, 32.19)	0.482
Divorced/widow^a^	2	50.0	2	50.0	1	
**Ethnicity**
Yoruba	146	45.5	175	54.5	1.05 (0.67, 1.6)	0.838
Non-Yoruba^a^	47	44.3	59	55.7	1	
**Religion**
Christianity	97	43.1	128	56.9	0.84 (0.57, 1.23)	0.360
Islamic^a^	96	47.5	106	52.5	1	
**No. of mother’s children**
1–4	185	45.2	224	54.8	1.03 (0.39, 2.67)	0.948
>4^a^	8	44.4	10	55.6	1	
**Child’s position**
First	64	47.4	71	52.6	1.13 (0.42, 3.03)	0.813
Second–fourth	121	44.2	153	55.8	0.99 (0.38, 2.58)	0.981
Beyond fourth^a^	8	44.4	10	55.4	1	–
**Mothers’ income level**
Low income	121	46.2	141	53.8	1.23 (0.45, 3.320)	0.689
Middle income	65	43.9	83	56.1	1.12 (0.40, 3.09)	0.829
High income^a^	7	41.7	10	58.8	1	–

*^a^Reference category; OR, odds ratio; CI, confidence interval*.

*p was considered significant if <0.06*.

### Types and prevalence of non-nutritive sucking

**Figure 1:**
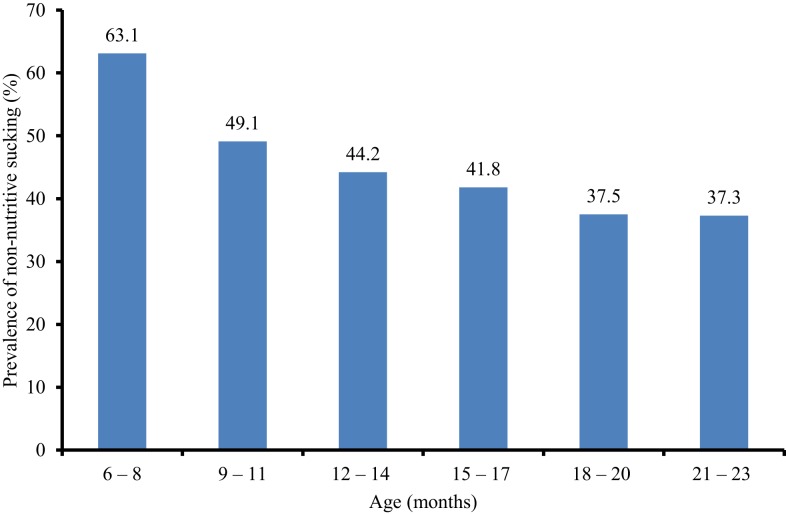
**Age-specific prevalence of non-nutritive sucking among children**.

Of the 427 mothers, 45.2% (193/427) reported that their children engaged in non-nutritive sucking habit. Further analysis showed that the prevalence of finger sucking, plastic nipple dummy, and both dummy and fingers was 31.3, 12.6, and 1.4%, respectively. The age-specific prevalence of non-nutritive sucking was as displayed in Figure 1. However, it was observed that the prevalence of non-nutritive sucking decreased significantly as the age increased (χ^2^ for trend = 17.857, *p* = 0.007). There was no significant difference between the prevalence of non-nutritive sucking among male (45.8%) and female (44.5%) children (χ^2^ = 0.071, *p* = 0.790).

Participants whose mothers had less than secondary education (45.7%) had the highest prevalence of non-nutritive sucking, compared with those whose mothers had secondary education (45.3%) and higher education (44.2%). However, the differences were not statistically significant. Similarly, children whose mothers were small scale traders (47.2%) had the highest prevalence of non-nutritive sucking, compared with those whose mothers were artisan (43.7%), civil servant/private firm workers (44.9%), and unemployed (41.7%) but the differences were not statistically significant.

### Acute diarrhea, non-nutritive sucking, and feeding practices

In all, 55.0% (235/427) of the mothers reported acute diarrhea episodes in their children, of which, users and non-users of non-nutritive sucking constituted 60.6 and 50.4%, respectively. The odds ratio of acute diarrhea was significantly higher among users than non-users of non-nutritive sucking (OR = 1.51; 95% CI = 1.03, 2.23; *p* = 0.035), after controlling for feeding methods. Of the 235 children who had episodes of diarrhea, 45.8% were males and 44.5% of females.

Table 2 shows the association between feeding techniques and non-nutritive sucking habits. The prevalence non-nutritive sucking habits was significantly higher among children who were not exclusively breastfed (159/297 = 53.5%) than those who were exclusively breastfed (34/130 = 26.2%). There was an increased odds ratio of non-nutritive sucking habits among children not exclusive breastfed compared with those who were exclusively breastfed (OR = 3.25; 95% CI = 2.07, 5.12). Further analysis revealed that children who were breastfed were less likely to have non-nutritive sucking habits compared with children whose breast feeding was timed (OR = 2.42; 95% CI = 4.88, 1.19). Also, bottle feeding was significantly associated with non-nutritive sucking habits compared with use of cup and spoon for feeding (OR = 3.16; 95% CI = 1.56, 6.40).

**Table 2:** **Feeding practices and non-nutritive sucking habit**.

Breastfeeding practices	Non-nutritive sucking				OR (95% CI)	*p*
	Yes		No	
	*n*	%	*n*	%	
**Exclusive breastfeeding**
Yes	34	26.2	96	73.8	0.307 (0.19, 0.48)	0.000
No^a^	159	53.5	138	46.5	1	–
**Breast feeding pattern**
On demand	169	43.3	221	56.7	0.414 (0.20, 0.83)	0.012
Timed^a^	24	64.9	13	35.1	1	–
**Complementary feeding methods**
Spoon/cup	106	38.4	170	61.6	0.98 (0.53, 1.84)	0.961
Bottle feeding	68	66.7	34	33.3	3.16 (1.56, 6.40)	0.001
Cup and spoon feeding^a^	19	38.8	30	61.2	1	–

*^a^Reference category; OR, odds ratio; CI, confidence interval*.

*p was considered significant if <0.06*.

### Hand washing and plastic dummy cleansing practices among mothers

Table 3 shows the distribution of mothers by their responses to questions on hand washing practices and whether or not their children engaged in non-nutritive sucking habit. There were no significant associations between whether or not mothers practiced hand washing often and non-nutritive sucking habit with respect to food preparation, child’s time of feeding, and defecation. Of the 54 mothers of children with plastic dummy sucking habit, 41 (75.9%) claimed they wash the plastic dummy with soap and water each time it dropped on the floor while 13 (24.1%) would pick it up and replace into child’s mouth.

**Table 3:** **Hand washing practices of mothers of children aged 6–23 months in the study area**.

	Non-nutritive sucking habit				p*
	Yes (193)		No (214)	
	*n*	%	*n*	%	
**Mother washes child’s hands at least twice daily**					
Often	18	9.3	16	6.8	0.344
Not often	175	90.7	218	93.2	
**Hand washing before preparing child’s food**					
Often	178	92.2	214	91.5	0.771
Not often	15	7.8	20	8.5	
**Hand washing before feeding the child**					
Often	140	72.5	188	80.3	0.057
Not often	53	27.5	46	19.7	
**Hand washing after defecation**					
Often	86	44.6	108	46.2	0.742
Not often	107	55.4	126	53.8	
**Hand washing after cleaning child’s perineum**					
Often	50	25.9	67	28.6	0.530
Not often	143	74.1	167	71.4	

*p was considered significant if <0.06.

## Discussion

This study revealed that prevalence of non-nutritive sucking habits among children aged 6–23 months in the study area was 45.2% and acute diarrhea was more frequent among those who engaged in non-nutritive sucking (60.6%) than those who did not (50.4%). The study showed that age, poor breastfeeding pattern, failure to practice exclusive breast feeding in the first 6 months of life, and inappropriate complementary feeding practices were significantly associated with non-nutritive sucking habits. Despite the different intervention programs toward the reduction of the global burden of childhood diarrhea diseases, the burden of this illness remains high in developing countries (5, 7, 8). The persistence of acute diarrhea among children aged 6–23 months in Nigeria may be related to the high prevalence of non-nutritive sucking habits.

The prevalence of non-nutritive sucking habits in the study population is comparable to 45.6% reported by Garbin et al. (23), and 40.2% reported by Santos et al. (24) but lower that 26.6% reported by Jahanbin and colleagues (25). The higher prevalence of non-nutritive sucking habits than reported by Jahanbin and colleagues (25) could be attributed to the fact that the present study participants were relatively younger. Moreover, the present study showed decreasing prevalence of non-nutritive sucking habits in children as age increases. This observation suggests that as the age increases the children tend to give up non-nutritive sucking habits. It is worth noting that the percentage of finger sucking (31.3%) found in our study was higher than plastic nipple dummy sucking (12.6%). This difference is expected, as it reflects the views of Bishara et al. (26), who stated that the finger is more accessible to children than the plastic nipple dummy and children will have greater trouble ceasing the finger-sucking habit compared to the plastic pacifier. It further reflects the notion that plastic nipple dummy use among children is a lesser common cultural practice among Nigerian population.

Another key finding from this study is that the prevalence of acute diarrhea episode among children with non-nutritive sucking habits was higher than those without. To the best of our knowledge, this is the first study that is reporting a significant association between non-nutritive sucking and acute diarrhea disease in Nigeria. It is essential to understand the extent non-nutritive sucking habit relates to the burden of acute diarrhea among children because of its well-known usage in the urban communities. However, two previous studies in Brazil (6, 21) found no significant association between acute diarrhea and non-nutritive sucking habit. One reason for the discrepancy in the findings could be the differences in the study designs. While our study was cross-sectional in design and relied on mothers’ ability to recall events that occurred within 3 months before they were interviewed, participants were prospectively followed up in those studies (6, 21). Moreover, those studies (6, 21) investigated only plastic nipple dummy sucking while our study considered both plastic nipple dummy and finger sucking. These two non-nutritive sucking materials play the same role of calming and pacifying children in the Nigerian context. The less than optimal hygiene practices of mothers whose children had non-nutritive sucking habits could explain the association between non-nutritive sucking habits and acute diarrhea (6).

In our study, exclusive breast feeding on demand protected against non-nutritive sucking habits in children. The proportion of children who had non-nutritive sucking habits was considerably higher among children not exclusively breastfed (53.5%) than those who were exclusively breastfed (26.2%). This finding agrees with the report of Aarts et al. (27) and Ngom et al. (4) but contrast with that of Jahanbin et al. (25) who opined that the finger-sucking habit tends to be high among exclusively breastfed children. In addition, our data revealed that complementary feeding pattern was significantly associated with non-nutritive sucking habits. Children who were bottle-fed were three times more likely to engage in non-nutritive sucking than those fed with cup and spoon. This observation agrees with reports by Yassaei et al. (28) and Maia-Nader et al. (29). In all, these findings suggest that good breastfeeding and weaning practices have the potential to help in reducing problems associated with non-nutritive sucking habit.

The findings from the present study have a number of implications. First, it has for the first time drawn attention to the need to consider advocacy and policy formulation toward reducing the use of pacifiers and finger sucking in the early phase of child development in Nigeria. Second, the data presented has built on existing evidence on the need to promote hand washing practices and emphasize health talks among nursing mothers as well as promotion of exclusive breast feeding at every opportunity. Third, the understanding of the association between pacifier attachment and diarrhea may guide policy, helps to design intervention programs that could help reduce childhood diarrhea episodes and the associated complications among Nigerian children.

Nonetheless, the findings of this study need to be interpreted within the limits of the validity of the instruments used to obtain information from respondents. Since the definition of acute diarrhea was solely based on mothers’ ability to recall events within 3 months prior to interview, it is not unlikely that there might have been some degree of recall bias. Also, the study did not include microbiological analysis, for possible isolation and identification of microbial etiological agents of diarrhea. Thus, one cannot be absolutely sure of the link between pacifier colonization and the incidence of acute diarrhea reported by the mothers. Another issue that cannot be absolutely argued from our data is that the lower prevalence of breastfeeding among children who engaged in non-nutritive sucking compared with those who did not was not responsible for the higher incidence of diarrhea, despite the use of logistic regression to adjust for confounders. Also, the fact that the study area was restricted to only the urban communities of south-western Nigeria limits the generalization of findings from the study. It may therefore be more informative to conduct a larger community-based prospective cohort study to validate the observed association between pacifier use (dummy and finger sucking) and childhood diarrhea episodes in Nigeria.

## Conclusion

This study has, for the first time, demonstrated a high prevalence of non-nutritive sucking habit among children <2 years old in urban communities in Nigeria. Diarrhea was more frequently reported among those who engaged in non-nutritive sucking than those who did not. It may be concluded that non-nutritive sucking habits such as plastic nipple dummy and finger sucking among children in the absence of proper hygiene practices poses an increased for acute diarrhea in children. It can also be deduced that child engagement in non-nutritive sucking habit did not influence hand washing practices of the mothers who participated in the study.

## Author Contributions

AO conceptualized and designed the study, carried out the data analyses, and drafted the manuscript. GO contributed to the design of the study, supervised data collection and entry, and contributed to preparation of the manuscript. Both authors read and approved final draft of the manuscript.

## Conflict of Interest Statement

The authors declare that the research was conducted in the absence of any commercial or financial relationships that could be construed as a potential conflict of interest.
